# Supplementary material for: MetaRibo-Seq measures translation in microbiomes
Source: Nat Commun. 2020 Jun 29;11:3268. doi: 10.1038/s41467-020-17081-z (PMC7324362; doi:10.1038/s41467-020-17081-z)
Supplement: Supplementary file 10 — Supplementary Data 7 [file 41467_2020_17081_MOESM10_ESM.zip › File2/Confidence_High_Taxonomy/123453_out.krona.html]

Javascript must be enabled to view this page.

members
magnitude
magnitudeUnassigned
count
unassigned
taxon
rank

123453\_out

3

superkingdom
2
3

phylum
976
3

class
200643
3

171549
order
3

family
815
3

genus
816
1

SRS147919\_contig\_number\_5779
3

1

SRS1041140\_contig\_number\_5224
47678
species

28116
species

SRS1055076\_contig\_number\_809
1
